# A review of penicillin binding protein and group A *Streptococcus* with reduced-β-lactam susceptibility

**DOI:** 10.3389/fcimb.2023.1117160

**Published:** 2023-03-31

**Authors:** Dingle Yu, Danchun Guo, Yuejie Zheng, Yonghong Yang

**Affiliations:** ^1^ Shenzhen Children’s Hospital, Shenzhen, China; ^2^ Microbiology Laboratory, National Center for Children’s Health, Beijing Pediatric Research Institute, Beijing Children’s Hospital, Capital Medical University, Beijing, China

**Keywords:** group A *Streptococcus* (GAS), *Streptococcus pyogenes*, antibiotic resistance, Penicillin binding protein, Pbp2x, reduced-penicillin susceptibility, β-lactam

## Abstract

With the widespread use of antibiotics, antimicrobial resistance (AMR) has become a global problem that endangers public health. Despite the global high prevalence of group A *Streptococcus* (GAS) infections and the global widespread use of β-lactams, β-lactams remain the first-line treatment option for GAS infection. β-hemolytic streptococci maintain a persistent susceptibility to β-lactams, which is an extremely special phenomenon in the genus *Streptococci*, while the exact current mechanism is not known. In recent years, several studies have found that the gene encoding penicillin binding protein 2X (*pbp2x*) is associated with GAS with reduced-β-lactam susceptibility. The purpose of this review is to summarize the current published data on GAS penicillin binding proteins and β-lactam susceptibility, to explore the relationship between them, and to be alert to the emergence of GAS with reduced susceptibility to β-lactams.

## Introduction

Group A *Streptococcus* (GAS), also known as *Streptococcus pyogenes* (*S. pyogenes*), is a very important human pathogen. With the widespread use of antibiotics, the incidence of GAS infection has decreased considerably, but it remains an important human pathogen, ranking in the top 10 causes in terms of morbidity and mortality of infectious diseases (Bessen et al., 2019), responsible for more than 700 million infection,1.8 million severe infections and 517,000 deaths worldwide each year (Carapetis et al., 2005; Ralph and Carapetis, 2013). Importantly, so far, there is no licensed vaccine to prevent GAS infections (Steer et al., 2016). GAS can cause a wide range of clinical conditions, from mild pharyngitis to life-threatening invasive infections (Brockmann et al., 2018; Lamagni et al., 2018; Liu et al., 2018).

According to the World Health Organization (World Health Organization, 2014), antimicrobial resistance (AMR) is a global challenge that poses a serious threat to public health and the world economy. In 2014, the WHO Global Antimicrobial Resistance Surveillance System (GLASS) released the first global report on AMR surveillance (World Health Organization, 2014). β-lactams are the of first-line antibiotics of choice for the treatment of most GAS infections. An anomaly in the biology of *S. pyogenes* is the persistent high susceptibility to β-lactams. To date, no naturally occurring penicillin-resistant strain in *S. pyogenes* has been identified (Horn et al., 1998; Suzuki et al., 2015; Chochua et al., 2017; Yu et al., 2020; Yu et al., 2021b). This is unusual because resistance to β-lactams has emerged independently several times in many other important Gram-positive human bacterial pathogens(Zapun et al., 2008). Resistance to β-lactams in *Streptococcus pneumoniae*, an important example of a Streptococcal pathogen, has been described globally. While rare, *Streptococcus agalactiae* (group B *Streptococcus* [GBS]) and *Streptococcus dysgalactia*e subspecies *equisimilis* (SDSE) reported *pbp2x* point mutations resulting in reduced susceptibility to β-lactams (Dahesh et al., 2008; Fuursted et al., 2016; Metcalf et al., 2017). In recent years, reduced-penicillin-susceptibility of GAS has been reported (Kimura et al., 2008; Metcalf et al., 2017). As previously observed in other streptococci, the emergence of mutations in certain PBP genes is considered a first step towards potential full penicillin resistance, and warrants continued surveillance (Jamin et al., 1993; Kimura et al., 2008; Hayes et al., 2020b).

## Interpretation criteria for antimicrobial susceptibility testing results of GAS to β-lactams

The Clinical and Laboratory Standards Institute (CLSI) and the European Commission on Antimicrobial Susceptibility Testing (EUCAST) guidelines are widely recognized and have long recommended penicillin for the treatment of GAS infections. There are no “intermediate” or “resistant” breakpoints to penicillin according to CLSI or EUCAST guideline. According to CLSI criteria, whose criteria have remained unchanged for many years, a minimum inhibition zone ≥24mm or a minimum inhibitory concentration (MIC)≤0.12 μg/mL for β-hemolytic streptococci indicates susceptibility to penicillin, and also to other β-lactams (amoxicillin, ampicillin and cefaclor).However, there have been some reports of GAS isolates being described as “non-susceptible” or “resistant” to β-lactams (Amábile-Cuevas et al., 2001; Capoor et al., 2006; Ogawa et al., 2011; Berwal et al., 2018). After reviewing these papers, we found that the terms “intermediate” or “resistant” used in these reports were not used accurately for interpretation of results (Yu et al., 2020; Yu et al., 2021b). The authors of these papers should have described the isolates as “non-susceptible” but instead referred to them as “intermediate” or “resistant”, while CLSI and EUCAST do not define breakpoints for these terms for GAS. Therefore, highlighting the current lack of understanding of GAS susceptibility breakpoints and interpretation for β-lactam antibiotics by many researchers.

## Penicillin and other β-lactams

β-lactam antibiotics, including penicillin, are a class of antibiotic molecules that disrupt bacterial cell walls during cell proliferation. They are fungal, natural or synthetic antimicrobial agents (Fleming, 1929; Sheehan and Henery-Logan, 1959). There are five classes of penicillin antibiotics including natural penicillins, aminopenicillins, penicillins resistant to penicillinase, extended-spectrum penicillins and aminopenicillin/β-lactamase inhibitor combinations (Miller, 2002). Other antibiotics also have typical β-lactam ring structures, including cephalosporins, carbapenems and monoamides, which together with penicillin are known as β-lactam antibiotics.

After the introduction of penicillin in the early 1940s, *Staphylococci* and *enterococci* (Miller et al., 2014) developed resistance within just a few years (Kirby, 1944; Lakhundi and Zhang, 2018). From the mid-1960s to the 1970s, intermediate strains of *Streptococcus pneumoniae* were sporadically reported (Hansman et al., 1974; Jacobs et al., 1979; Klugman, 1990). While still rare, from the mid-1990s, reports of reduced susceptibility to GBS has been documented (Kimura et al., 2008; Gaudreau et al., 2010). GBS strains with reduced susceptibility to β-lactams are been described in Japan and the United States (Dahesh et al., 2008; Seki et al., 2015; Kobayashi et al., 2021). SDSE is the species most closely related to GAS (Oppegaard et al., 2017). During 2010 to 2012, four incidents of penicillin-resistant (PR) SDSE isolated from blood cultures of three patients were detected in Denmark (Fuursted et al., 2016).

However, there are exceptions. A key exception to Fleming ‘s warning about the relationship between antimicrobial use and the development of resistance is the persistent susceptibility of GAS to β-lactam antibiotics (Horn et al., 1998). Eighty years after the introduction of penicillin, GAS strains still maintain consistent susceptibility to various β-lactams, and even the MICs of GAS to β-lactams remain low and stable (Yu et al., 2021a). The reasons for the persistent susceptibility of GAS to β-lactams are unclear, but may include differences in the rate and mechanism of horizontal gene transfer between GAS and other *Streptococci*.

## Mechanism of resistance

There are three main mechanisms of resistance to β-lactams, including destruction of the antibiotic by β-lactamases, reduced affinity for PBP binding, or reduced access to PBPs (Ambler, 1980). Resistance of Gram-positive organisms to β-lactams is mainly due to target modifications, in which PBPs undergo structural changes (Fisher and Mobashery, 2016). Reduced susceptibility to penicillin in GAS has been demonstrated due to amino acid substitutions within PBPs that affect the ability to bind penicillins (Jamin et al., 1993; Kimura et al., 2008; Hayes et al., 2020b).

## Mutations within PBPs

Identification and subsequent genetic analysis of antimicrobial resistant strains revealed that resistant strains have chimeric high molecular mass penicillin-binding proteins (HMM PBPs) compared to susceptible strains (Zighelboim and Tomasz, 1980; Dowson et al., 1989). In resistant Streptococci, the evolution from penicillin susceptible to reduced susceptibility and then to non-susceptible occurs through the progressive accumulation of amino acid substitutions in HMM PBPs rather than through single-event horizontal gene transfer of β-lactamase or low β-lactam affinity HMM PBPs (Zighelboim and Tomasz, 1980; Barcus et al., 1995; Kimura et al., 2008; Zapun et al., 2008; Fuursted et al., 2016). Thus, the identification of the genetic polymorphism in pathogenic streptococci leading to reduced antibiotic susceptibility has been observed from phenotype to genotype. This phenotype-to-genotype workflow has dominated the molecular basis of antibiotic resistance research for decades and is responsible for the discovery of novel mechanisms. GAS with reduced susceptibility to β-lactams have acquired mutations in genes encoding PBPs, including PBP1a, PBP2a, PBP2b and PBP2x. Some mutations have been identified, but the most common mutation in the GAS results in the substitution of amino acid in PBP2x transpeptidase for T553K (Vannice et al., 2020; Chochua et al., 2022) (**Table 1**).

**Table 1:** Reports of reduced susceptibility to β-lactams amongst group A *Streptococcus* isolates and the associated amino acid substitutions identified.

Country (study period)	Sample source	Number OR Rates	β-lactam antibiotics	Antibiotic resistance test	MIC	Breakpoint standards	Mutations identified					References
							*PBP2x*	*PBP2a*	*PBP2b*	*PBP1a*	*PBP1b*	
Mexico 2001	Pharyngotonsillitis	10(5%)	Penicillin G	E-test	0.25–0.75 μg/ml	NCCLS	—	—	—	—	—	(Amábile-Cuevas et al., 2001)
India 2002-2003	Acute pharyngotonsillitis	7(20.6%)	Penicillin	E-test	0.19-0.25μg/ml	CLSI	—	—	—	—	—	(Capoor et al., 2006)
Japan 2006-2008	Pharyngitis	2/93	Penicillin G	Broth microdilution	>2.0μg/ml	CLSI 2007	—	—	—	—	—	(Ogawa et al., 2011)
India 2016-2017	Upper respiratory tract infections	4(8%), 2(4.2%), 2(5.3%)	Ampicillin cefotaxime ceftriaxone	MALDI-TOF Mass Spectrometry (VITEK MS, bioMerieux)	MIC, 0.12-8μg/ml	CLSI	—	—	—	—	—	(Berwal et al., 2018)
Australia 2020	—	4/9667	NC	NC	NC	NC	STMK to SAMK STMK to STIK	—	—	—	—	(Hayes et al., 2020a)
USA 2017-2018	Blood and wound isolates	2/282	ampicillin & cefotaxime	Broth Microdilution & E-test	Ampicillin 8-fold higher, cefotaxime 3-fold higher	CLSI	T553K substitution	—	—	—	—	(Vannice et al., 2020)
USA 2020	Mouse Model of Necrotizing Myositis	—	—	—	—	—	Pro601Leu amino acid replacement in PBP2X	—	—	—	—	(Olsen et al., 2020)
Iceland 1995-2006	—	*emm*12 strains, 332/1575	A 2-fold increased penicillin G and ampicillin MIC	Disk diffusion method	—	CLSI, EUCAST	Met593Thr Ile502Val Pro676Ser Lys708Glu	—	—	—	—	(Southon et al., 2020)
Japan 2015-2016	Skin, Pus, Tonsil	3	—	Agar dilution method	—	CLSI	M593T, A397V	T459A	—	—	—	(Ikeda et al., 2021)
2022	—		—	—	—	—	Identified 464 *pbp2x* alleles	564 *pbp2a* alleles	—	389 *pbp1a* alleles	427 *pbp1b* alleles	(Beres et al., 2022)
USA 2015-2021	—	55/13727	None isolates exhibited nonsusceptibility to b-lactams	—	—	—	*emm*43.4/PBP2x-T553K variant, two isolates ampicillin MIC 0.25 mg/ml, 129/340 (37.9%) of isolates with elevated β-lactam MICs	—	—	—	—	(Chochua et al., 2022)
USA 2022	An isogenic mutant strain was generated and virulence assessed in a mouse model of necrotizing myositis	—	—	—	—	—	Strains with the chimeric SDSE-like PBP2X had reduced susceptibility *in vitro* to nine β-lactam antibiotics	—	—	—	—	(Olsen et al., 2022)

NCCLS (National Committee for Clinical Laboratory Standards) is the predecessor of Clinical and Laboratory Standards Institute (CLSI) ; WGS, Whole-genome sequencing; MALDI-TOF, Matrix Assisted Laser Desorption/Ionization-Time of Flight Mass Spectrometry; —, No data OR No clear.

## Prevalence of GAS with reduced-β-lactam susceptibility

All reports of GAS with reduced-β-lactam susceptibility worldwide to date are detailed in **Table 1**. Detailed characterization of GAS with reduced β-lactams susceptibility were first reported in 2001 (Amábile-Cuevas et al., 2001). Since then, it has also been reported in India (Capoor et al., 2006; Berwal et al., 2018), Japan (Ogawa et al., 2011; Ikeda et al., 2021), in Iceland(Southon et al., 2020) and in the United States (Musser et al., 2020; Vannice et al., 2020; Chochua et al., 2022).

In 2020, Hayes et al. investigated the relative frequency of PBP sequence variations in 9,667 *S. pyogenes* isolates worldwide (Hayes et al., 2020a). The majority of these genomic sequences were derived from UK and US datasets that focused on invasive diseases (Ben Zakour et al., 2015; Davies et al., 2015; Athey et al., 2016; Chalker et al., 2017; Chochua et al., 2017; Kapatai et al., 2017; Turner et al., 2017; Bergin et al., 2018; Coelho et al., 2019; Davies et al., 2019; Dickinson et al., 2019; Lynskey et al., 2019). They found that mutations in *S. pyogenes* PBPs occured rarely in this global database, with less than 3 amino acid changes differing in more than 99% of the world population. Only 4 of 9 667 strains contained mutations near the active sites of PBP2x or PBP1a transpeptidase. No reported PBP2x T553K mutation was found. Their findings imply that while heavy antibiotic pressure may select for mutations in the PBPs, there is currently no evidence that such mutations become fixed in the *S. pyogenes* population or that mutations in the PBPs are being sequentially acquired. However, because low levels of resistance to subclinical lactams could theoretically confer a biological advantage to GAS, vigilance in monitoring population GAS for PBP mutations is encouraged (Musser et al., 2020).

In 2022, Beres et al. (Beres et al., 2022) analyzed 26,465 *S. pyogenes* genome sequences. Population genomic data identified amino acid changes in PBP1a, 1b, 2a, and 2x. The evolutionary signature of these proteins under positive selection is a potential candidate for reduced susceptibility to β-lactams. In 2022, Olsen et al. (Olsen et al., 2022) again noted that whole genome sequencing identified a GAS strain containing a chimeric PBP2x derived from an SDSE recombinant fragment. The results suggest that mutations such as PBP2x chimeras may lead to reduced susceptibility to β-lactams and increased fitness and virulence.

Hanage et al. (Hanage and Shelburne, 2020) noted that studies have shown that mutations of PBP are associated with reduced susceptibility of *S. pneumoniae* to β-lactam antibiotics (Li et al., 2016), *Streptococcus agalactiae* (Dahesh et al., 2008a), some SDSE(Fuursted et al., 2016) and GAS (Vannice et al., 2020).In another study (Musser et al., 2020), two related *Streptococcus pyogenes* strains with reduced susceptibility to ampicillin, amoxicillin, and cefotaxime, antibiotics commonly used to treat *S. pyogenes* infections, were reported. The two strains had the same nonsynonymous (amino acid-substituting) mutation in the *pbp2x* gene, encoding penicillin- binding protein 2X (PBP2X). They identified 137 strains that together had 37 nonsynonymous mutations in 36 codons of *pbp2x*. The authors propose that GAS with reduced susceptibility to β-lactams associated with mutations in the *pbp2x* gene are geographically widespread. Does this study suggest that we are finally at the beginning of the era of widespread susceptibility of GAS to β-lactams? They believed that this answer is no (Hanage and Shelburne, 2020).

## Summary

Currently, penicillin, the first-line treatment of GAS infection, is generally considered effective for GAS. However, reports of reduced susceptibility to β-lactams are becoming more common. For now, clinicians can continue to be confident that β-lactams remain the agents of choice for the treatment of GAS infections. The fluctuating nature of the emergence of GAS strains, including those with reduced susceptibility to various antimicrobials, means that ongoing surveillance of the GAS population is both in the public health interest and helps clinicians understand the changing nature of medically important bacteria.

## Author contributions

YZ and YY proposed the topic of this review. DY and DG conducted a literature search and wrote this review. YZ and YY revised and edited the manuscript. All authors contributed to the article and approved the submitted version.
